# Health matters: a statistical approach to understand childhood illnesses in the North-East States of India, 2019–2021

**DOI:** 10.1186/s12889-024-20090-y

**Published:** 2024-11-12

**Authors:** Mukesh Ranjan, Lalrotlinga, Ashutosh Singh, Brajesh, Vanlalhriatsaka

**Affiliations:** 1 Department of Statistics, Pachhunga University College, Mizoram University, Aizawl, Mizoram 796001 India; 2 Department of Geography, Pachhunga University College, Mizoram University, Aizawl, Mizoram 796001 India; 3 Department of Geography, Hemwati Nandan Bahuguna Garhwal University (HNBGU), SRT Campus, Tehri, Uttarakhand 249145 India; 4 Department of Statistics, Kishinchand Chellaram College, Mumbai, Churchgate 400020 India

**Keywords:** North eastern Indian states, Childhood illnesses, Diarrhoea, ARI, Fever, NFHS-5

## Abstract

The present study explores the prevalence and socio-economic demographic factors affecting childhood illnesses. Diarrhoea, fever and ARI among under-five children in the North -East states of India using NFHS-5 data Kids file. Results showed that diarrhoea, ARI, and fever among the northeastern states were highest in Meghalaya.For diarrhoea Sikkim has the highest prevalence for children within 6 months while Meghalaya has the highest prevalence in the age groups 6- 12 months and 1- 2 years old children and Arunachal Pradesh has the highest rate in the age group 2- 5 years old children. Meghalaya stands out with the highest prevalence of fever and ARI in all age groups. Compared to Sikkim, the state of Meghalaya had more diarrhoea, ARI and fever, and it was statistically highly significant. However, Tripura and Assam had significantly higher odds of having fever and ARI than Sikkim. There is an association between diarrhoea, fever, and ARI and factors such as the age of the child and caregiver, the wealth status of the household, the quality of sanitation facilities, methods of stool disposal, and the caregiver's educational level.

## Introduction

Globally, diarrhoea ranks as the second leading cause of death among children under 5 years old, accounting for 1.5 million fatalities annually worldwide [1]. In India, it stands as the third leading cause of childhood mortality [1]. A cross-sectional study conducted across 707 districts in India, encompassing 223,785 children, reveals that 7.3 percent of children in the country suffer from diarrhoea [2]. An examination conducted among children under 3 years old in India through NFHS-5 revealed that out of 132,198 children surveyed, 8.4 percent had experienced diarrhoea in the two weeks leading up to the study. Among those lacking improved toilet facilities, the prevalence rose to 12.5%, whereas it stood at 9.7% among those utilizing improved facilities and highlight the significance of enhanced sanitation in reducing diarrhoea [3]. A study conducted in Nepal in 2019 identified that children between 7–23 months old, those experiencing Acute Respiratory Infection (ARI), and children whose mothers lacked access to prenatal care faced a heightened risk of developing diarrhoea [4]. Childhood diarrhoea presents a significant public health challenge in India, contributing to 14% of deaths among children under five. Oral Rehydration Solution and zinc are effective in managing diarrhoea [5]. According to the 2017 educational and health survey conducted in Bangladesh, approximately 5% of children reported having episodes of diarrhoea [6]. In India diarrhoea is prevalent among children from scheduled caste, rural and economically disadvantage families [7]. Environmental factors, including child stool disposal practices, and the materials used for household roofs and floors emerged as significant contributors to childhood diarrhoea [8]. The decomposition analysis conducted in Peru revealed a pro-poor inclination. Children aged 0–23 months from the lowest wealth indexes in the highland and jungle regions emerged as notable contributors [9]. In Ethiopia, the findings indicated lower odds of childhood illness when the mother's age fell within the range of 35–49 years compared to those aged 15–24 and attributed to older mothers' experience in child and health management [10]. Zinc supplements have been identified as a remedy for diarrhoea and respiratory illnesses [11].

The United Nations Sustainable Development Goals (UN SDGs) emphasizes to end preventable deaths of children under age 5 years by 2030 among all world countries including India [12]. Globally, Acute respiratory Infections (ARI), fever, and diarrhea are the prominent causes of the burden of childhood diseases along with mortality mostly in developing and low resource setting countries [13, 14].Even in India, children under age 5 years are suffering from and contributing a significant amount to the total disease burden of children under age 5 which are due to the childhood diseases of diarrhoea, ARI and fever [15–20]. Even these three childhood Illnesses depend upon some factors and needs intervention strategy to reduce the burden of preventable deaths due to these Illnesses [15, 17–24]. The prevalence of the three preventable childhood Illnesses viz Diarrhoea, fever and ARI in North-east Indian states were also high and noticed by some of the previous studies [16, 20, 25–27].Diarrheal illness represents a major worldwide public health concern and a leading cause for hospitalization among under 5 children and detoriation in quality of life on a global level [28]. Globally, sufficient medical care has been given to diarrhoea and acute respiratory infections (ARI),however they remain among the prime cause of mortality in children under five years old [1, 29]. Respiratory infections (ARI), fever and diarrhoea play a substantial role in the worldwide disease burden and mortality in developing countries [30, 31]. Numerous cases of diarrhoea remain undiagnosed, due to their mild symptoms and tend to resolve on their own, or because those affected choose not to seek medical assistance [32].

Diarrhoea stands as one of the top five causes of death for infants and children under the age of five in India [33]. Every year, millions of children under five years old worldwide lose their lives to environment-related illnesses such as Acute Respiratory Infections and acute diarrhoea [34, 35]. Extreme dehydration and the loss of fluids stand as the key factors contributing to fatalities caused by diarrhoea, impacting both adults and children [36]. Despite a decrease in the overall mortality rate for children under 5, diarrhoea continues to be a substantial factor, causing an estimated 300,000 child deaths annually in India [37] Around 400,000 children under 5 years old succumb to Acute Respiratory Infection (ARI)-related ailments annually [38–40]. A recent study based on NFHS-4 found, among the northeastern region, the state of Meghalaya performs worst in terms of diarrhoea and ARI [25].Even in NFHS-5 report, diarrhoea and ARI in Meghalaya was much higher than the national prevalence of the illnesses [7].

A study on diarrhoea in North-East India, utilizing data from DLHS-4, observed that a 61.46% of women continued normal breastfeeding, while 56.63% provided their children experiencing diarrhoea with Zinc or ORS. [41]. The Northeastern region of India, comprising of the seven sister states (Mizoram, Tripura, Assam, Manipur, Meghalaya, Arunachal Pradesh, Nagaland) as well as the Himalayan states of Sikkim, showcases diversity characterized by unique cultural, geographical, and environmental aspects [42–44]. The unique blend of climatic conditions, limited healthcare accessibility, and dense forest coverage in select areas render this region vulnerable to infectious Illnesses [42].Waterborne illnesses such as cholera, diarrhoea and typhoid prevail in this region due to inadequate sanitation facilities, low awareness levels, and limited access to safe drinking water [45]. In the DLHS-3 baseline, households show a 35 percent adoption of piped water and a 36 percent adoption of improved sanitation. There are noticeable discrepancies in coverage across various states. Piped water coverage is below 15 percent in northeastern states, especially low in Assam. Conversely, Arunachal Pradesh and Sikkim demonstrate piped water coverage rates exceeding 85 percent. Additionally, Mizoram displays improved sanitation coverage rates surpassing 80 percent [46]. Addressing infectious Illnesses in Northeast India presents a range of complex challenges. These include inadequate healthcare infrastructure, scarce trained professionals and limited accessibility to healthcare in remote areas. These obstacles hinder effective management and control of Illnesses. Socio-economic factors such as illiteracy, poverty and insufficient awareness further increase the population's susceptibility to infectious Illnesses. The government has undertaken numerous strategies in India to mitigate diarrhoea and enhance public health. These initiatives include improving access to clean water and sanitation facilities, advocating for proper hygiene practices, and promoting oral rehydration therapy for timely treatment. Additionally, the government has emphasized emphasized emphasized vaccination programs, particularly against rotavirus, a primary cause of diarrheal Illnesses in children. Ongoing endeavors involve a blend of policies, awareness campaigns, and enhancements to healthcare infrastructure to tackle these challenges persistently.

Limited research has been carried out to understand the prevalence and factors affecting the three childhood Illnesses in north east Indian states. The present study aims to estimate the prevalence, association, and socio-economic demographic and health factors affecting childhood disease illnesses, diarrhoea, fever, and ARI among under-five children in the North-East states of India.

### Data source

The data used in this study was unit level data of the fifth round of National Family Health Survey data (NFHS-5) conducted in 2019–21.NFHS-5 provides the information on population, health and nutrition for India and its each state and union territories and district level estimates for many important indicators. The NFHS also provides information on crucial parameters concerning population demographics, family planning, maternal and child health, nutritional status, adult health, and instances of domestic violence, among other factors. The data is available from National Family Health Survey (NFHS-5) (rchiips.org). NFHS-5 fieldwork for India was conducted in two phases, phase one from 17 June 2019 to 30 January 2020 and phase two from 2 January 2020 to 30 April 2021 and gathered information from 636,699 households, 724,115 women, and 101,839 men. The birth file of NFHS-5 contains the information of nearly 1,274,250 children who were between 1978 to 2021.The information about each of these children along with their survival status, year of birth, sex of the child, birth order, age at death, whether a child is twin or not and other information was asked retrospectively to all the 724,115 eligible women who were ever married in the age group 15–49 years.

In this study we used the five year truncated birth history file which is popularly known as Kids file where the information for each of the children were given for past 5 years before the survey period, so here the information details about the children was asked from 2014 onwards till the year 2019–2021. Information about health and these Illnesses among the children are available for the past five years however for the vaccination and other details the information was asked for the children born in the past three years before the survey year (i.e. from 2016 onwards).

Hence in Kids file,232,920 children were nested among 176,843 ever married women.Since our study was based on the north east Indian states hence we selected the 34,222 children of all eight north east Indian states ( i.e. Sikkim, Assam,Arunachal Pradesh, Nagaland,Manipur,Mizoram,Meghalaya, Tripura).For our study for understanding the childhood Illnesses like diarrhoea,fever and Acute Respiratory Infections(ARI) we have taken only alive children which consists of 33,199 children. So the final sample for our study was 33,199 children whose information was collected in for northeast Indian states in the past five years.

The definitions for ARI, Diarrhoea used in the study were based on questions asked in NFHS from the child’s mother/respondent during the survey. The acute respiratory infection, ARI symptoms consist of cough accompanied by (1) short, rapid breathing that is chest related, and/or (2) difficult breathing that is chest related. Mothers who reported about their children under age five years who had symptoms of acute respiratory infection (ARI) in the two weeks before the survey. This question is asked only if the child had a cough in the past two weeks. Short, rapid breathing or difficulty breathing are signs of pneumonia or other acute respiratory infections, which are a principal cause of death among children (IIPS,2019).Similary Diarrhoea was defined for children under age five years with diarrhoea in the two weeks before the survey. It has considered a child with diarrhoea as those children who passed ‘three or more loose or liquid stools per day.’ while reading this question, emphasize ‘in the last 2 weeks’ [47] to reduce the response from recall bias as far as possible and also reflect the disease conditions [48–50] The question related to fever was “Has the child been ill with a fever at any time in the last 2 weeks?”.

The reponse for the three illnesses were not recorded during the same month or season for all the northeast states as the survey was conducted in phases. For example, NFHS-5 fieldwork for Arunachal Pradesh was conducted from January 2020 to March 2020 before the lockdown and from December 2020 to April 2021 post lockdown, similarly fieldwork for Assam was conducted from June, 2019 to December, 2019, for Manipur fieldwork was conducted from July, 2019 to January, 2020, for Meghalaya it was conducted between July, 2019 to November, 2019, for Mizoram from July, 2019 to November, 2019,for Nagaland it was conducted from July, 2019 to December, 2019, fieldwork for Sikkim was conducted from August, 2019 to December, 2019 and finally fieldwork for Tripura was conducted from 4 July, 2019 to 10 November, 2019 respectively.In present study, we have not considered the seasonality factor for the considered illnesses even if there is any.

In the NFHS-5, the information about diarrhoea was asked for each birth in 2014 or later. The question was asked from mothers about her under 5 years children about childhood illnesses diarrhoea as “Has had diarrhoea in the last 2 weeks” and the response was recorded in “yes” and “no”. Similarly, the questions related to fever and ARI was asked for its occurrence in the last two weeks. For our study for each of the three childhood Illnesses (like Diarrhoea, fever and ARI) we have coded “0” if the illnesses has not occurred in the past two weeks and “1” if it occurred in the past two weeks. We have estimated the prevalence and association of these three childhood Illnesses by various community characteristics (like states, place of residence) Mother or Individual characteristics like (age, education, religion, caste), Household Characteristics (wealth index, sanitation facilities, water facilities, number of children living), child characteristics (sex of the child, birth order, child’s age, caesarean delivery, stunting).

For logistic regression, each of these dichotomous childhood diseases was taken as the dependent variable, and the different community, household, individual, and child characteristics were taken as the independent variable and included in the model as dummy variables.

The kids file of NFHS-5 was used for this objective which has the information of about 33,199 alive children who were born in the year 2014 or later and the information about diarrhoea was collected from them but for vaccination since the information was collected from the year 2016 onwards, so the sample size of the children get reduced to 19,089 children. We used separate logistic regression model for examining the factors associated with diarrhoea for the two samples in the form of two models i.e. Model 1 & Model 2 respectively. Model 1 was based on 33,199 children and Model 2 based on 19,089 children when vaccination was included in model 1 as shown in Table 5.

#### Dependent and independent variables used in the logistic regression

In the three separate logistic regressions, three different dependent variables, viz., having diarrhoea, fever, and ARI in the past two weeks before the survey, were taken as dependent variables, with “0” indicating the absence of the disease illnesses and “1” indicating their presence.

The choice governing the selection of the independent variables was based on the previous literature and the variables at least show some association with the dependent variable. The independent variables or covariates in Model 1 were kept into broad categories as community, household, individual,and child variables. The community level variables were i.e. state and place of residence, which was included as dummy variables with Sikkim as the reference category for state and place urban as reference for place of residence. In household characteristics, type of toilet facilities variable was recoded into two categories as 1 for “improved sanitation” and 0 for “unimproved sanitation” facilities. Under improved sanitation following categories of the type of toilet facility i.e. flush to piped sewer system, flush to septic tank, flush to pit latrine, ventilated improved pit latrine (vip), pit latrine with slab and composting toilet was coded as 1 and rest all of its other categories were included under unimproved category coded as “0″.Number of living children was into three categories coded as “one child”, “two children” and “3 or more children”. Similarly for the individual characteristics, Mothers age in 5 years was recategorized into three categories “15–24″,”25–34″ and “35–49″ years; Caste was categorised into “SC”,”ST”, “Obc” and “Others”; Religion into 4 categories “Hindu”,”Muslim”,”Christian” and “others”. In child characteristics birth order was categorized into four categories one birth order “1 BO”, two birth order “2 BO”,third birth order as “3 BO” and 4 or more birth order as “4 plus BO”; the z scores for height/age standard deviation variable was recategorized into two categories into stunted “yes” and not stunted “ no" as per WHO guideline; child’s age in months was computed from the subtraction of two variables ( date of survey in century month code (cmc) and date of birth of child in cmc)) and recategorized into four categories “within 6 months”, “6–12 months”,”1 to less than 2 years” and “2 to 5 years” and caesarean section included as dummy variable. In Model 2,we have included programme variables like BCG,DPT and Rota virus apart from those in Model 1. In programme factors each of the factors BCG,DPT and Rota virus vaccination was used as independent variables in its dichotomous form with vaccination date on card and vaccination marked on the card was kept under yes category and all others were kept under no category.

## Methods

### Logistic regression

Statistical analysis were performed using Stata version 15. Bivariate analyses involving cross-tabulations were conducted independently for each disease Illnesses(diarrhea, fever and ARI). These analyses aimed to establish connections between the occurrence of childhood illnesses and various selected variables, which would later be employed in the multivariate analysis. Significant determinants were explored using Pearson's Chi-square (χ^2^) test. For the multivariable analysis, logistic regressions were employed to assess the impact of explanatory variables on the probability of childhood illness. This approach was crucial in identifying significant risk factors associated with fever, ARI (acute respiratory infection), and diarrhea in children. To maintain accuracy, three distinct binary logistic regression models were employed for each illnesses separately, without considering any confounding factors.

The logistic regression model is expressed as:

$$\text{Pr }(Yi = 1) =\frac{\mathit{exp}\left({X}_{i}\beta \right)}{1+\mathit{exp}\left({X}_{i}\beta \right)}$$

where, *Y*_*i*_ is a binary variable that takes a value of ‘1’ if the respondent has illnesses and ‘0’ otherwise, X_i_ is a vector of independent variables and $\beta$ is a vector of unknown parameters.

The estimated form of regression is define as

$$\text{In}\left[\frac{{\widehat{P}}_{i}}{1-{\widehat{P}}_{i}}\right]={\widehat{\beta }}_{0}+{\widehat{\beta }}_{1}{X}_{1}+\dots +{\widehat{\beta }}_{k}{X}_{k}$$

The odds ratio in favor of *Y*_*i*_ = 1 was computed for *X*_1_,*X*_2_…*X*_*k*_ to indicate how often the group of interest is more likely to be associated with the child's illnesses than the reference group.

Table 1 shows the total sample size and information about categorical variables converted in to dummy variables used for running the logistic regression model. We see that there are missing cases in some of the dummy variable which is from the original data. But the result of this study will still give an admissible results.

**Table 1:** Dummy variables descriptive statistics as used in the logistic regression model

Variables (#)	Observations	Mean	Std. Dev	Min	Max
**Overall North East Indian States**					
Diarrhoea	33,199	0.06	0.2378	0	1
Fever	33,199	0.151	0.3579	0	1
Acute Respiratory Infections	32,953	0.025	0.1554	0	1
**North East States**					
Arunachal Pradesh	33,199	0.164	0.3699	0	1
Nagaland	33,199	0.089	0.2843	0	1
Manipur	33,199	0.095	0.2926	0	1
Mizoram	33,199	0.073	0.2593	0	1
Tripura	33,199	0.06	0.2375	0	1
Meghalaya	33,199	0.193	0.3943	0	1
Assam	33,199	0.31	0.4624	0	1
**Residence**					
Rural	33,199	0.85	0.3568	0	1
**Mother's Age**					
25- 34	33,199	0.563	0.4961	0	1
35–49	33,199	0.175	0.3803	0	1
**Education**					
Primary	33,199	0.173	0.3781	0	1
Secondary	33,199	0.591	0.4917	0	1
Higher	33,199	0.078	0.2677	0	1
**Religion**					
Muslim	33,199	0.146	0.353	0	1
Christian	33,199	0.466	0.4989	0	1
Others	33,199	0.092	0.2891	0	1
**Caste**					
SC	28,341	0.083	0.2751	0	1
ST	28,341	0.705	0.4561	0	1
Obc	28,341	0.125	0.3306	0	1
**Wealth Index**					
Poorer	33,199	0.315	0.4645	0	1
Middle	33,199	0.18	0.3843	0	1
Richer	33,199	0.099	0.2986	0	1
Richest	33,199	0.032	0.1751	0	1
**Sanitation Facilities**					
Sanitation Unimproved	32,890	0.253	0.4346	0	1
**Birth Order**					
2	33,199	0.295	0.4561	0	1
3	33,199	0.152	0.3586	0	1
4 plus	33,199	0.183	0.3865	0	1
**Stunting status**					
Yes	31,395	0.35	0.4769	0	1
**Child's Age**					
6–12 months	33,199	0.113	0.3167	0	1
1–2 years	33,199	0.17	0.3753	0	1
2- 5 years	33,199	0.631	0.4826	0	1
**Living Children**					
2	33,199	0.331	0.4707	0	1
3_more	33,199	0.37	0.4828	0	1
**Delivery**					
Cesearan delivery	33,199	0.137	0.3434	0	1
**Immunization Taken**					
Bcg	19,089	0.788	0.4087	0	1
Dpt	19,089	0.613	0.4871	0	1
Rota virus	19,089	0.269	0.4435	0	1

## Results

The study sample comprised of 33,199 children age under 5 years old in the North East states of India drawn from the National Family Health Survey-5 (NFHS-5). All statistical analyses were based on the three common childhood illnesses variables such as diarrhoea, fever and ARI (Table 2 below). The children of both sexes are nearly same in the sample. The age distribution of children reveals nearly 17 percent aged 1 to 2 years, 63 percent aged 2 to 5 years while remaining 20 percent are less than 1 years of age. Cesarean deliveries accounted for nearly 18 percent of childbirths. Nearly, 35 percent of the children experienced stunting. Health-wise, 5 percent children suffered diarrhoea, 2.51 percent had Acute Respiratory Infection (ARI) and 17.36 percent had fever. Of the total children, children from Assam notably contributed 70 percent in sample, followed by Meghalaya (10.32%), Tripura (7.14%), and respectively varying contributions from Mizoram( 2.17%), Manipur(5.39%), Nagaland (2.54%), Arunachal Pradesh(2.09%), and Sikkim(0.74%). The percentage of children reported here are the weighted percentage and hence they are representative one since the sampling weights are adjustment factors applied to each case in tabulations to adjust for differences in probability of selection and interview between cases in a sample, due to either design or happenstance (IIPS & ICF,2021). Rural areas accounted for 85 percent children. The percentage of children belonging to mothers of different age groups in the sample comprises nearly one-third of children born to mothers in 25–29 age group, more than 20 percent born to 20–24 years and 30–34 years women age group respectively. Educationally, 15 percent of children born to illiterate women,17 percent children belonged to mothers who completed primary education, 61 percent children born of mothers who completed secondary education, while nearly 7 percent children belonged to mothers who attained higher education. The majority of children (45.32%) in the sample born to mothers who were Hindus, followed by Muslim children (33.34%), Christian (18.36%), and other religions (3%). Nearly 40 percent children had mothers belonged to Scheduled Castes, 27 percent children belonged to OBCs, Scheduled Tribes children comprises of nearly 16 percent, and remaining castes nearly 17 percent. Financially, 72.4 percent of children hailed from impoverished backgrounds (poorest and poorer one), 12 percent of children belonged to wealthy families (richer and richest), and 16 percent to middle class families. Children born to households with Improved water facilities were prevalent (83%), yet sanitation facilities lagged (34% only). Children born to families with one, two, and three children comprised 36 percent, 36 percent and 29 percent, respectively, of the total children in the surveyed households.

**Table 2:** Study Sample description for Northeast Region of India, 2019–2021

Variables/Characteristics	Percentage	N
***Childhood Illness***		
** Diarrhoea**		
No	94.05	31,201
Yes	5.95	1,998
** ARI**		
No	97.49	32,137
Yes	2.51	816
** Fever**		
No	82.64	28,190
Yes	17.36	5009
***Community Characteristics***		
** State**		
Sikkim	0.74	609
Arunachal Pradesh	2.09	5,430
Nagaland	2.54	2,945
Manipur	5.39	3,140
Mizoram	2.17	2,406
Tripura	7.14	1,992
Meghalaya	10.32	6,392
Assam	69.62	10,285
** Place of Residence**		
Rural	85.16	28,227
Urban	14.84	4972
***Mother's Characteristics***		
** Age**		
15–19	4.29	1087
20–24	27.81	7614
25–29	33.94	11,185
30–34	20.26	7490
35–39	10.47	4223
40–44	2.57	1222
45–49	0.66	378
** Education**		
Illiterate	15.13	5264
Primary	16.51	5739
Secondary	61.01	19,617
Higher	7.35	2579
** Religion**		
Hindu	45.32	9814
Muslim	33.34	4844
Christian	18.36	15,484
Others	3	3057
** Caste**		
SC	15.71	2338
ST	40.35	19,979
OBC	27.02	3539
Others	16.91	2485
***Household Characteristics***		
** Wealth Index**		
Poorest	40.57	12,427
Poorer	31.85	10,456
Middle	15.61	5980
Richer	8.8	3285
Richest	3.17	1051
** Sanitation Facilities**		
Not improved	65.8	24,578
Improved	34.2	8312
** Water facilities**		
Not improved	17.05	5981
Improved	82.95	27,218
** Number of living children**		
one child	35.61	9919
two child	35.84	11,001
three or more children	28.56	12,279
***Child Characteristics***		
** Sex of the child**		
Male	50.71	16,816
Female	49.29	16,383
** Birth Order**		
1 BO	41.62	12,304
2 BO	31.93	9794
3 BO	13.36	5031
4 plus BO	13.1	6070
** Child's Age**		
within 6 months	8.66	2875
6–12 months	11.35	3753
1to less than 2 years	17.35	5630
2 to 5 years	62.64	20,941
** Caesarean delivery**		
No	82.27	28,666
Yes	17.73	4533
** Stunting**		
No	64.84	20,411
Yes	35.16	10,984
**Total**	**100**	**33,199**

From Table 3, it is observed that within the overall sample data, the prevalence of diarrhoea shows a high association with living in each of the North-East states of India. The place of residence whether rural or urban showed a statistically highly significant (*p* < 0.01) relationship with diarrhoea among under 5 children. Age of the mother was statistically significantly (*p* < 0.05) associated with prevalence of diarrhoea among children Similary, her educational level, religion, caste were all statistically highly significantly (*p* < 0.01) associated with the prevalence of diarrhoea among children. The wealth index of the family, sanitation facilities, were statistically highly significantly (*p* < 0.05) associated with the prevalence of diarrhoea among children while the water facilities was not associated statistically. Sex of the children and no. of living children in the family did not show any statistically significant relationship with childhood diarrhoea. In contrast, birth order of the child, stunted growth, child’s age and the type of delivery of the child were statistically significantly highly associated with diarrhoea.

**Table 3:** The association of Diarrhoea, Fever and ARI with various community, household, individual and child characteristics for under-5 children in North-East Indian States, 2019–21

	**Diarrhoea**		**Chi-square**	**Fever**		**Chi-square**	**ARI**		**Chi-square**
	**No**	**Yes**		**No**	**Yes**		**No**	**Yes**	
***Community characteristics***									
** State/Region**									
Sikkim	578 (572.3)	31(36.7)	𝜒^2^ = 238.4128, *p* < 0.01	528(517.1)	81(91.9)	𝜒^2^ = 839.0456, *p* < 0.01	596(588.1)	7(14.9)	𝜒^2^ = 201.8196, *p* < 0.01
Arunachal Pradesh	5119(5103.20)	311(326.8)		4966(4610.7)	464(819.3)		5266(5258.5)	5266(133.5)	
Nagaland	2830(2767.80)	115(177)		2692(2500.7)	253(444.3)		2888(2846.7)	31(72.3)	
Manipur	2986(2951)	154(189)		2762(2666.2)	378(473.8)		3063(3063.9)	51(77.1)	
Mizoram	2324(2261)	82(144.8)		2215(2043)	191(363)		2377(2329.8)	12(59.2)	
Tripura	1864(1872)	128(119.9)		1641(1691)	351(300.5)		1941(1924.1)	32(48.9)	
Meghalaya	5761(6007.30)	631(384.7)		4856(5427.6)	1536(964.4)		6053(6187.9)	292(147.1)	
Assam	9739(9666)	546(619)		8530(8733.2)	1755(1551.8)		9953(9965)	265(253)	
** Place of Residence**									
Rural	26,469(26,528.2)	1758(1698.8)	𝜒^2^ = 14.6711, *p* < 0.01	23,904(23,968.2)	4323(4258.2)	𝜒^2^ = 7.6022, *P* < 0.01	27,310(27,328.1)	712(693.9)	𝜒^2^ = 3.2367, *p* = 0.072
Urban	4732(4672.8)	240(299.2)		4286(4221.8)	686(750.2)		4827(4808.9)	104(122.1)	
***Mothers characteristics***									
** Age**									
15–19	1004(1021.6)	83(65.4)	𝜒^2^ = 16.1010, *p* < 0.05	888(923)	199(164)	𝜒^2^ = 29.5204, *P* < 0.01	1061(1059.1)	25(26.9)	𝜒^2^ = 9.0340, *p* = 0.172
20–24	7107(7155.8)	507(458.2)		6392(6465.2)	1222(1148.8)		7393(7399.1)	194(187.9)	
25–29	10,533(10,511.9)	652(673.1)		9490(9497)	1695(1687.6)		10,815(10,828.1)	288(274.9)	
30–34	7066(7039.2)	424(450.8)		6457(6359.9)	1033(1130.1)		7263(7233.3)	154(183.7)	
35–39	3994(3968.8)	229(254.2)		3605(3585.8)	618(637.2)		4065(4078.4)	117(103.6)	
40–44	1143(1148.5)	79(73.5)		1024(1037.6)	198(184.4)		1179(1175.2)	26(29.8)	
45–49	354(355.3)	24(22.7)		334(321)	44(57)		361(363.8)	12(9.2)	
** Education**									
Illiterate	4928(4947.2)	336(316.8)	𝜒^2^ = 59.0396, *p* < 0.01	4533(4469.8)	731(794.2)	𝜒^2^ = 29.5539, *p* < 0.01	5073(5095.6)	152(129.4)	𝜒^2^ = 23.9420, *p* < 0.01
Primary	5295(5393.6)	444(345.4)		4787(4873.1)	952(865.9)		5509(5548.1)	180(140.9)	
Secondary	18,494(18,436.4)	1123(1180.6)		16,615(16,657.2)	3002(2959.8)		19,038(18,992.7)	437(482.3)	
Higher	2484(2423.8)	95(155.2)		2255(2189.9)	324(389.1)		2517(2500.5)	47(63.5)	
** Religion**									
Hindu	9305(9223.4)	509(590.6)	𝜒^2^ = 18.6605, *p* < 0.01	8340(8333.3)	1474(1480)	𝜒^2^ = 64.1915, *p* < 0.01	9537(9500.8)	205(241.2)	𝜒^2^ = 17.6036, *p* < 0.01
Muslim	4536(4552.5)	308(291.5)		3941(4113.1)	903(730.9)		4660(4695.8)	155(119.2)	
Christian	14,513(14,552.1)	971(931.9)		13,246(13,147.8)	2238(2336.2)		14,993(14,985.5)	373(380.5)	
Others	2847(2873)	210(184)		2663(2595.8)	394(461.2)		2947(2955)	83(75)	
** Caste**									
SC	2223(2197.7)	115(140.3)	𝜒^2^ = 13.4294, *p* < 0.01	2032(1996.3)	306(341.7)	𝜒^2^ = 56.5506, *p* < 0.01	2265(2264.2)	55(55.8)	𝜒^2^ = 12.4418, *p* < 0.01
ST	18,717(18,779.9)	1262(1199.1)		17,202(17,059.1)	2777(2919.9)		19,322(19,342.6)	497(476.4)	
OBC	3340(3326.6)	199(212.4)		2900(3021.8)	639(517.2)		3423(3428.6)	90(84.4)	
Others	2360(2335.9)	125(149.1)		2065(2121.8)	420(363.2)		2438(2412.6)	34(59.4)	
***Household characteristics***									
** Wealth Index**									
Poorest	11,560(11,679.1)	867(747.9)	𝜒^2^ = 54.3417, *p* < 0.01	10,392(10,552)	2035(1875)	𝜒^2^ = 65.6077, *p* < 0.01	11,974(12,039.3)	371(305.7)	𝜒^2^ = 31.8976,*p* < 0.01
Poorer	9832(9826.7)	624(629.3)		8801(8878.4)	1655(1577)		10,124(10,121)	254(257)	
Middle	5647(5620.1)	333(359.9)		5188(5077.7)	792(902.3)		5804(5778.3)	121(146.7)	
Richer	3146(3087.3)	139(197.7)		2888(2789.4)	397(495.6)		3209(3182.2)	54(80.8)	
Richest	1016(987.7)	35(63.3)		921(892.4)	130(158.6)		1026(1016.2)	16(25.8)	
** Sanitation Facilities**									
Not improved	7735(7811.4)	577(500.6)	𝜒^2^ = 16.5842, *p* < 0.01	6855(7058.5)	1457(1253.5)	𝜒^2^ = 52.0629, *p* < 0.01	7997(8043.1)	252(205.9)	𝜒^2^ = 14.1374, *p* < 0.01
Improved	23,174(23,097.6)	1404(1480)		21,075(20,871.5)	3503(3706.5)		23,833(23,786.9)	563(609.1)	
** Water facilities**									
Not improved	5618(5621)	363(360)	𝜒^2^ = 0.0335, *p* = 0.855	5083(5078.6)	898(902.4)	𝜒^2^ = 0.0308, *p* = 0.861	5791(5796.8)	153(147.2)	𝜒^2^ = 0.2871, *p* = 0.592
Improved	25,583(25,580)	1635(1638)		23,107(23,111.4)	4111(4106.6)		26,346(26,340.2)	663(668.8)	
***Child characteristics***									
** Sex of the child**									
Male	15,805(15,804)	1011(1012)	𝜒^2^ = 0.0023, *P* = 0.962	14,301(14,278.8)	2515(2537.2)	𝜒^2^ = 0.4621, *p* = 0.497	16,248(16,283.5)	449(413.5)	𝜒^2^ = 6.3499, *p* < 0.05
Female	15,396(15,397)	987(986)		13,889(13,911.2)	2494(2471.8)		15,889(15,853.5)	367(402.5)	
** Birth Order**									
1 BO	11,598(11,563.5)	706(740.5)	𝜒^2^ = 23.4181, *P* < 0.01	10,482(10,447.6)	1822(1856.4)	𝜒^2^ = 47.9692, *p* < 0.01	11,948(11,907.6)	262(302.4)	𝜒^2^ = 41.5336, *p* < 0.01
2 BO	9229(9204.6)	565(589.4)		8380(8316.3)	1414(1477.7)		9506(9489.1)	224(240.9)	
3 BO	4750(4728.2)	281(302.8)		4342(4271.9)	689(759.1)		4877(4864.5)	111(123.5)	
4 plus BO	5624(5704.7)	446(365.3)		4986(5154.2)	1084(915.8)		5806(5875.8)	219(149.2)	
** Stunting**									
No	19,223(19,171.8)	1188(1239.2)	𝜒^2^ = 6.4269, *p* < 0.05	17,385(17,298.8)	3026(3112.2)	𝜒^2^ = 8.0515, *p* < 0.01	19,817(19,764.2)	455(507.8)	𝜒^2^ = 16.1130, *p* < 0.01
Yes	10,266(10,317.2)	718(666.8)		9223(9309.2)	1761(1674.8)		10,579(10,631.8)	326(273.2)	
** Child Age**									
within 6 months	2755(2702.0)	120(173)	𝜒^2^ = 109.5894, *p* < 0.01	2558(2441.2)	317(433.8)	𝜒^2^ = 176.9798, *p* < 0.01	2819(2803.8)	56(71.2)	𝜒^2^ = 17.1754, *p* < 0.01
6–12 months	3426(3527.1)	327(225.9)		3011(3186.8)	742(566.2)		3639(3660.1)	114(92.9)	
1to less than 2 years	5198(5291.2)	432(338.8)		4587(4780.6)	1043(849.4)		5461(5490.6)	169(139.4)	
2 to 5 years	19,822(19,680.7)	1119(1260.3)		18,034(17,781.5)	2907(3159.5)		20,218(20,182.5)	477(512.5)	
** C-section delivery**									
No	26,985(26,940.8)	1,681(1,725.2)	𝜒^2^ = 8.8218, *p* < 0.01	24,452(24,340.9)	4214(4325.1)	𝜒^2^ = 24.6019, *p* < 0.01	27,793(27,745.5)	657(704.5)	𝜒^2^ = 24.0263, *p* < 0.01
Yes	4,216(4,260.2)	317(272.8)		3,738(3849.1)	795(683.9)		4344(4391.5)	159(111.5)	
** No. of children living**									
one child	9,291(9,322.0)	628(597.0)	𝜒^2^ = 5.6483, *p* = 0.059	8286(8422.4)	1633(1496.6)	𝜒^2^ = 25.3133, *p* < 0.01	9,648(9632.4)	229(244.6)	𝜒^2^ = 18.3698, *p* < 0.01
two child	10,386(10,338.9)	615(662.1)		9463(9341.2)	1538(1659.8)		10,679(10,637.9)	229(270.1)	
three or more	11,524(11,540.0)	755(739.0)		10,441(10,426.4)	1838(1852.6)		11,810(11,866.7)	358(301.3)	

*Note the no. in the bracket() indicate the estimated frequency*

From Table 3 further, it is evident that within the overall sample data, prevalence of fever showed a statistically highly significant (*p* < 0.01) association with the states of North-East India. The place of residence, age of the mother, education level, religion she belongs to, caste and wealth index were also statistically highly associated with fever. Water facilities was not associated while sanitation facilities and stool disposal was significantly associated with fever. Sex of the child was not associated with fever. In contrast, the birth order of the child, stunting growth, age of the child and the number of children living in the family was statistically highly associated with fever.

Furthermore, From Table 3 we notice that from the overall sample data ARI was highly associated with each of the North-East states of India. Children living in the rural and urban had not much association with ARI. The mother age had no association with the ARI illnesses but education of the mother was highly associated with ARI on their children. Religion, Caste, family wealth, sanitation facilities and stool disposal had association with ARI.While water facilities had no association with ARI. Sex of the child was associated with 0.05% level of significance. Birth order of the child, stunting growth, the age of the child and the number of children were also associated with ARI.

From Table 4, we found that among the northeastern states, prevalence of diarrhoea in Meghalaya was nearly 10 percent followed by Tripura with 6 percent. In contrast, the lowest prevalence lied in the state of Nagaland of with 3.4 percent children suffered diarrhoea. Except Mizoram where the prevalence of diarrhoea among children was 4.3 percent, rest all four states had diarrhoea nearly 5 percent. For the entire northeastern region, the prevalence was nearly 6 percent and all proportions were statistically highly significant (*p* < 0.01). Prevalence of diarrhoea was higher in rural area (6.2%) than urban areas (4.3%). Among the children, the prevalence of diarrhoea among both male and female under 5 years children were nearly 6 percent. Although they were statistically highly significant, there was not much difference in prevalence of diarrhoea among stunted and not stunted children (*p* < 0.01). Among the different age group children, the prevalence of diarrhoea was highest among the children in the age group of 6–12 months (9.2%) followed by children of 1 to less than 2-year-old (7.1%), then among the children of 2 to 5 years old (5.4%) and least among children of less than 6 months (3.7%).The prevalence of diarrhoea was higher among children who were delivered not by cesearean (6.2%) than those who were delivered by cesearean (4.5%). Mother in age group 45–49 shows that the prevalence of diarrhoea is highest among their children (7.2%) and for other age group women the prevalence of diarrhoea among their children was nearly 6 percent. It was observed that among the mother who completed their primary education, the prevalence of diarrhoea among their children was nearly 8 percent and among the children of illiterate women the prevalence of diarrhoea was 6.7 percent. The lowest prevalence of diarrhoea was observed among children of higher educated mother. Among religion the prevalence of diarrhoea among Hindu children was 4.8 percent followed by Muslim (6.5%) and among Chistian and other religion children it was nearly 7 percent.

**Table 4:** The prevalence of diarrhoea, fever and ARI among under-5 years children by different socio-economic and demographic characteristics in North-East India, 2019–2021

Variables/Characteristics	Diarrhoea	SE	Fever	SE	ARI	SE
***Community characteristics***						
** State/Region**						
Sikkim	5.5***	1.386	18.1***	2.566	0.7**	0.344
Arunachal Pradesh	5.1***	0.324	9.0***	0.468	2.1***	0.208
Nagaland	3.4***	0.372	8.8***	0.643	1.1***	0.22
Manipur	5.5***	0.539	13.0***	0.775	1.7***	0.299
Mizoram	4.3***	0.664	10.1***	0.973	0.6***	0.226
Tripura	6.2***	0.574	16.9***	0.923	1.3***	0.249
Meghalaya	10.4***	0.546	22.9***	0.697	4.8***	0.387
Assam	5.5***	0.261	17.7***	0.441	2.5***	0.172
** North-East India**	5.9***	0.198	17.4***	0.327	2.51***	0.129
** Place of Residence**						
Rural	6.2***	0.222	16.4***	0.843	2.54***	0.141
Urban	4.3***	0.388	17.5***	0.355	2.31***	0.317
***Mother’s characteristics***						
** Age**						
15–19	6.3***	0.921	18.8***	1.717	2.8***	0.602
20–24	6.6***	0.408	19.1***	0.687	2.41***	0.263
25–29	5.5***	0.324	18.0***	0.561	2.75***	0.241
30–34	6.1***	0.447	14.8***	0.65	2.01***	0.239
35–39	5.3***	0.561	15.3***	0.868	2.31***	0.335
40–44	5.8***	1.051	16.8***	1.804	3.62***	0.451
45–49	7.2***	2.289	16.3***	3.606	1.21**	2.399
** Education**						
Illiterate	6.7***	0.55	16.6***	0.812	3.11***	0.367
Primary	7.9***	0.569	17.9***	0.81	3.26***	0.377
Secondary	5.6***	0.242	17.8***	0.426	2.28***	0.157
Higher	3.1***	0.509	13.9***	1.072	1.43***	0.319
** Religion**						
Hindu	4.8***	0.274	16.4***	0.484	1.86***	0.164
Muslim	6.5***	0.413	18.9***	0.671	3.16***	0.289
Christian	7.5***	0.35	17.4***	0.473	2.93***	0.226
Others	7.4***	0.742	15.1***	1.024	8.99***	0.389
** Caste**						
SC	4.1***	0.529	14.4***	0.966	1.91***	0.34
ST	6.7***	0.31	16.2***	0.426	2.79***	0.19
OBC	5.6***	0.461	18.9***	0.809	2.28***	0.296
Others	5.2***	0.585	17.2***	1.021	4.04***	0.284
***Household characteristics***						
** Wealth Index**						
Poorest	7.2***	0.354	18.1***	0.53	3.03***	0.231
Poorer	5.8***	0.345	18.1***	0.576	2.54***	0.222
Middle	5.1***	0.427	16.5***	0.815	1.9***	0.282
Richer	3.2***	0.449	14.3***	1.027	1.5***	0.311
Richest	2.1***	0.546	12.7***	1.589	1.27***	0.447
** Sanitation Facilities**						
Not improved	6.5***	0.385	19.4***	0.628	3.01***	0.25355
Improved	5.6***	0.227	16.4***	0.379	2.28***	0.1479
** Water facilities**						
Not improved	6.0***	0.479	16.4***	0.732	2.78***	0.328
Improved	5.9***	0.217	17.6***	0.364	2.45***	0.14
***Child characteristics***						
** Sex of the child**						
Male	6.0***	0.282	17.31***	0.456	2.82***	0.192
Female	5.9***	0.277	17.42***	0.469	2.18***	0.172
** Birth Order**						
1 BO	5.2***	0.283	17.5***	0.527	2.11***	0.19
2 BO	5.8***	0.353	17***	0.578	2.49***	0.228
3 BO	6.0***	0.558	16.16***	0.836	2.65***	0.375
4 plus BO	8.7***	0.631	19.03***	0.844	3.67***	0.387
** Stunting**						
No	5.8***	0.253	17.56***	0.424	2.27***	0.159
Yes	6.3***	0.347	17.78***	0.568	3***	0.242
** Child Age**						
within 6 months	3.7***	0.486	11.78***	1.004	2.11***	0.408
6–12 months	9.2***	0.705	22.41***	1.042	3.09***	0.388
1to less than 2 years	7.1***	0.518	19.99***	0.822	3.02***	0.345
2 to 5 years	5.4***	0.241	16.49***	0.406	2.31***	0.159
** Delivery by C-section**						
no	6.2***	0.224	17.34***	0.357	2.6***	0.147
yes	4.5***	0.409	17.44***	0.809	1.9***	0.26
** No. of children living**						
One child	5.6***	0.322	18.8***	0.59	2.1***	0.208
two child	5.4***	0.324	16.4***	0.538	2.3***	0.205
three or more	6.9***	0.39	16.6***	0.558	3.2***	0.266

^***^ means *p* < 0.01

^**^ means *p* < 0.05

There was a greater chance of children being influence by diarrhoea among Schedule Tribe, children belonged to the poorest family and the household which have no improved sanitation and water facilities.

The data from Table 4 further indicated a statistically significantly higher prevalence of fever in Meghalaya (23%) and lowest in Arunachal Pradesh (9%). In most cases, urban areas showed a higher prevalence than rural areas. Among children, female exhibited a notably higher prevalence with fever than male and children with stunted growth. Children aged 6–12 months have high prevalence with fever, and most were born with the caesarean section birth. Mothers in the 15 to 19 years and 20–24 age group had children with higher fever prevalence than other age groups. Additionally, mothers who have completed up to primary or secondary education showed relatively higher likelihood of prevalence of fever among their children. The majority of children with higher prevalence of fever belonged to the Muslim religion and were part of the OBC. Children from the poorest families were notably more prone to fever and often lacked improved sanitation while water facilities are improved.

Data from Table 4 furthermore, highlighted the statistically significantly higher prevalence of ARI was found in Megalaya (4.8%) followed by Assam (2.5%),Arunachal Pradesh (2.1%) while in the states like Mizoram and Sikkim it was less than 1 percent.In most cases, rural areas exhibited a higher prevalence of ARI than urban areas. Among children, males showed a significantly higher prevalence with ARI than females and most children with stunted growth. Children aged 6–12 months and those children born with not cesearean delivery were linked to higher ARI. Prevalence of ARI was highest among the children of mothers with the 40–44 age group. Furthermore, mothers who completed primary education had a higher prevalence of ARI to their children.Among religion other than Hindu, Muslim and Christian the prevalence of ARI was highest i.e. 9 percent. Similarly among the children of Other caste women,the prevalence of ARI was 4 percent and higher than ST,SC and OBCs. Children from the poorest families were notably more prone to ARI and often lacked improved sanitation and water facilities.

**Fig. 1:**
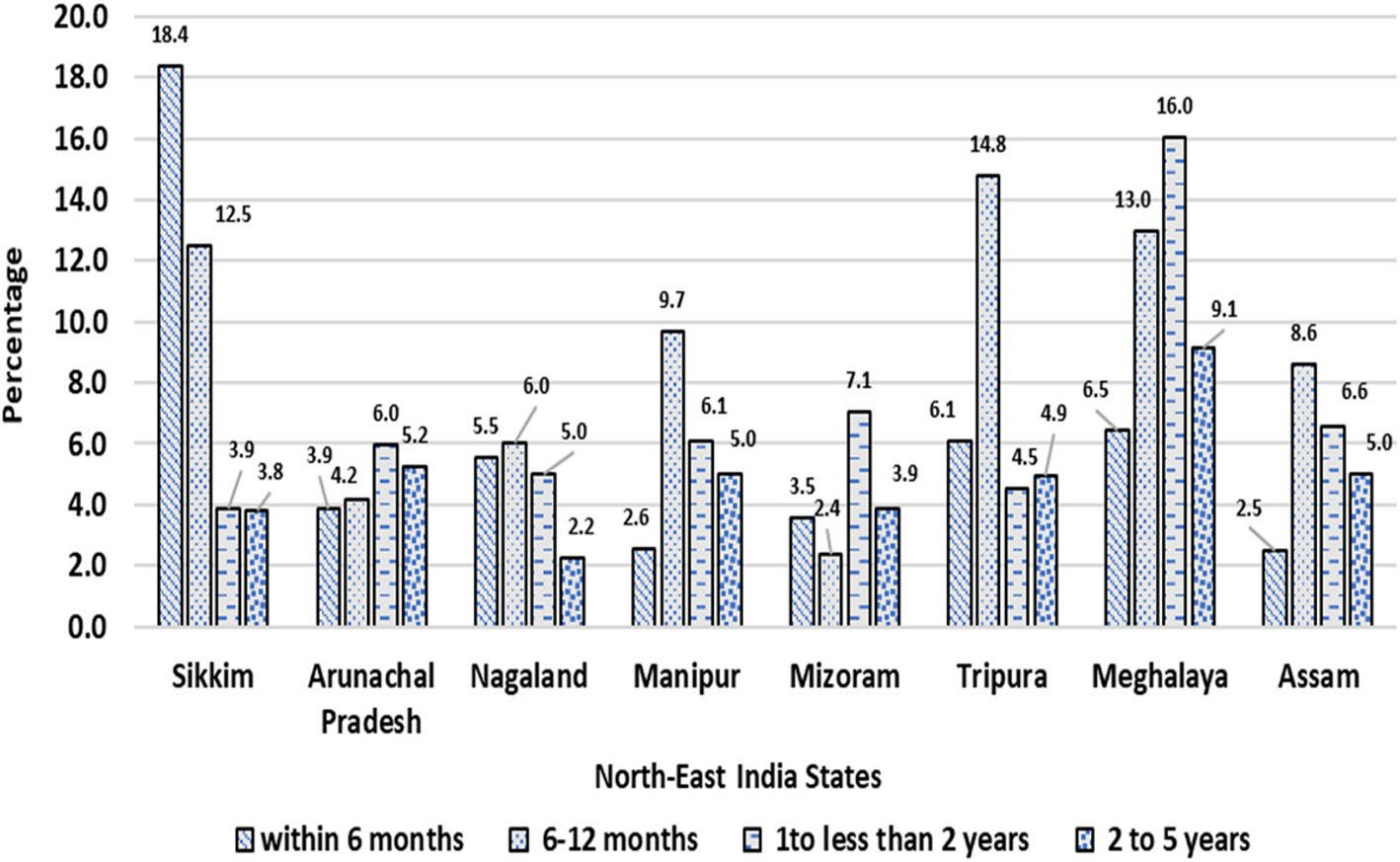
Prevalence of diarrhoea by age groups of under-5 children in North-East Indian States,India,2019–21

**Fig. 2:**
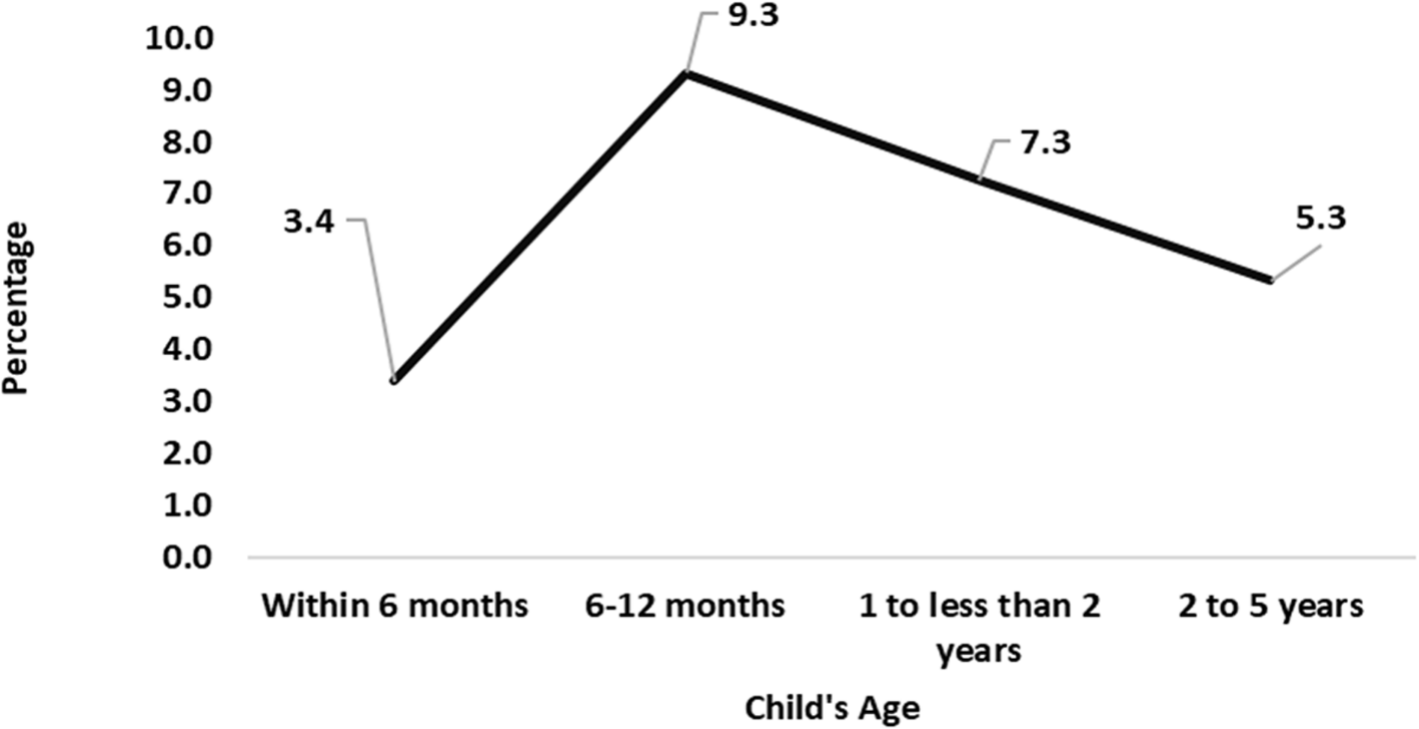
Prevalence of diarrhoea by age groups of under-5 children in North-East region,India,2019–21

In Figs. 1 and 2 show the prevalence of diarrheoa by different age group of children in state wise and the entire north east states of India. we see that Sikkim has the highest prevalence of diarrhoea in the age group within 6 months; Meghalaya has the highest prevalence in the age group 6- 12 months and 1- 2 years old children; Arunachal Pradesh has the highest rate in the age group 2- 5 years old children. We also observed that within the entire north east states 6 -12 months old children have highly prevalence with diarrhoea.

**Fig. 3:**
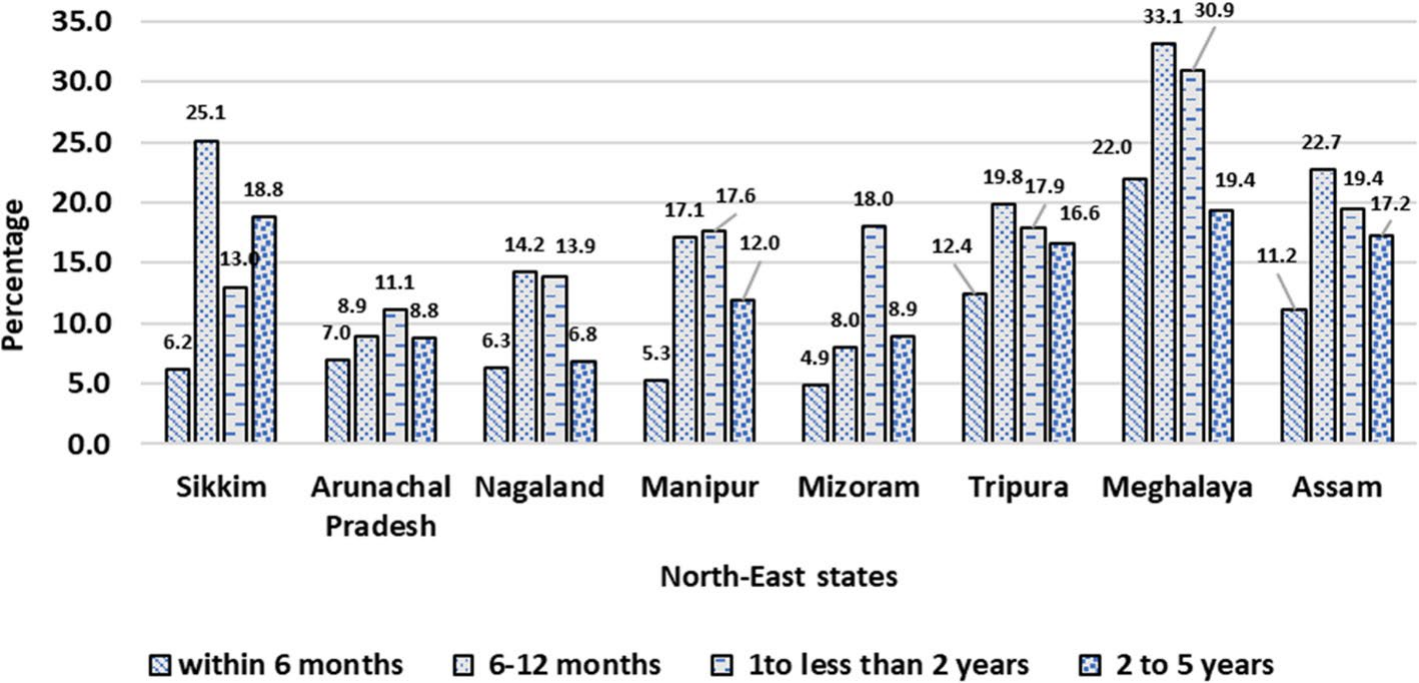
Prevalence of fever by age groups of under-5 children in North-East Indian States, India,2019–21

**Fig. 4:**
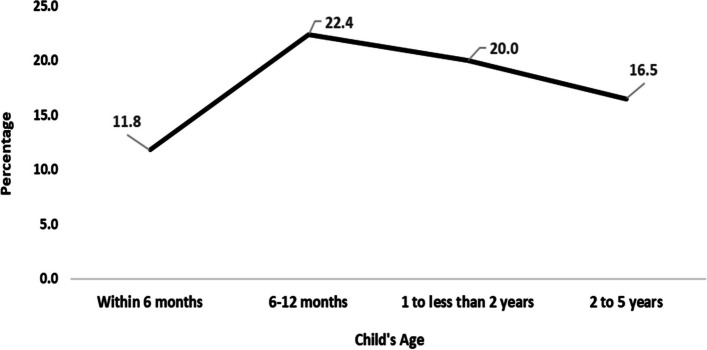
Prevalence of fever by age groups of under-5 children in North-East region,India,2019–21

In Figs. 3 and 4 depict the prevalence of fever among children across different age groups in both individual states and the entire Northeastern region of India. Meghalaya stands out with the highest prevalence of fever within the 6-month age group. Meghalaya exhibits the highest prevalence in children aged 6–12 months and 1–2 years old, at the same time Meghalaya records the highest rates among children aged 2–5 years. A noticeable trend across all the Northeastern states is the heightened prevalence of fever among children aged 6–12 months.

**Fig. 5:**
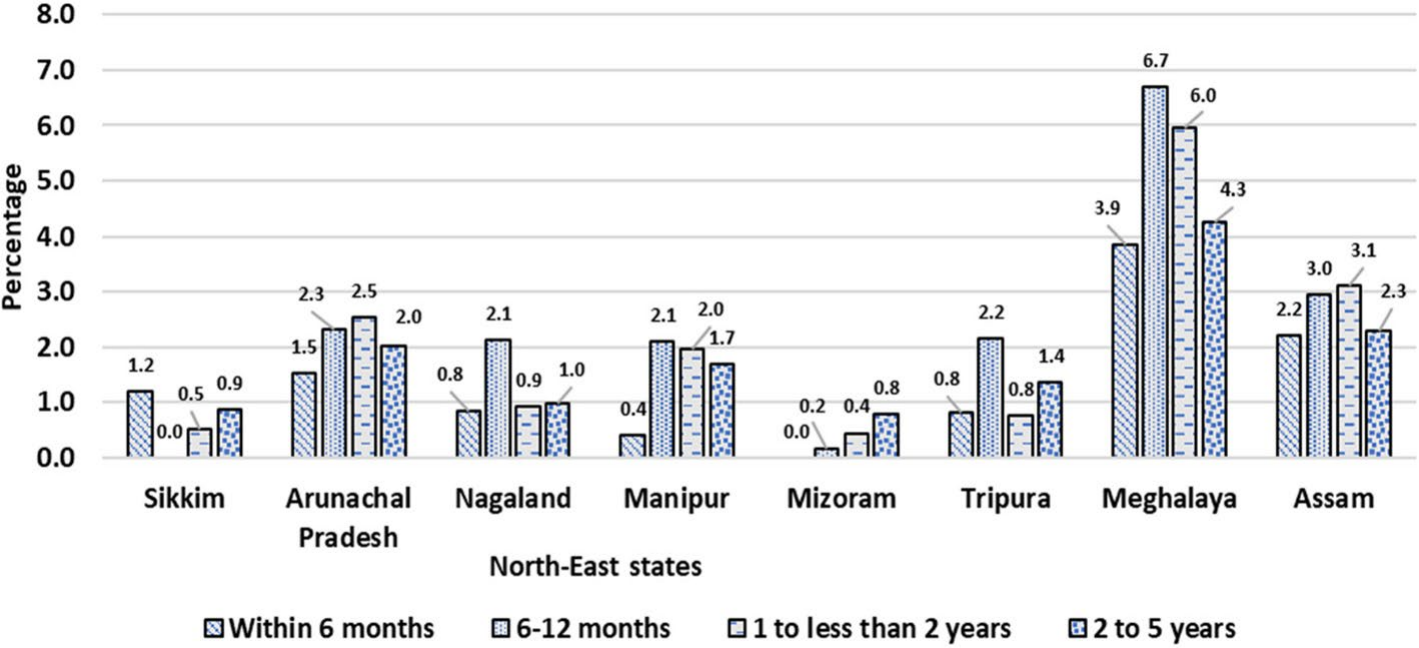
Prevalence of ARI by age groups of under-5 children in North-East Indian States, India,2019–21

**Fig. 6:**
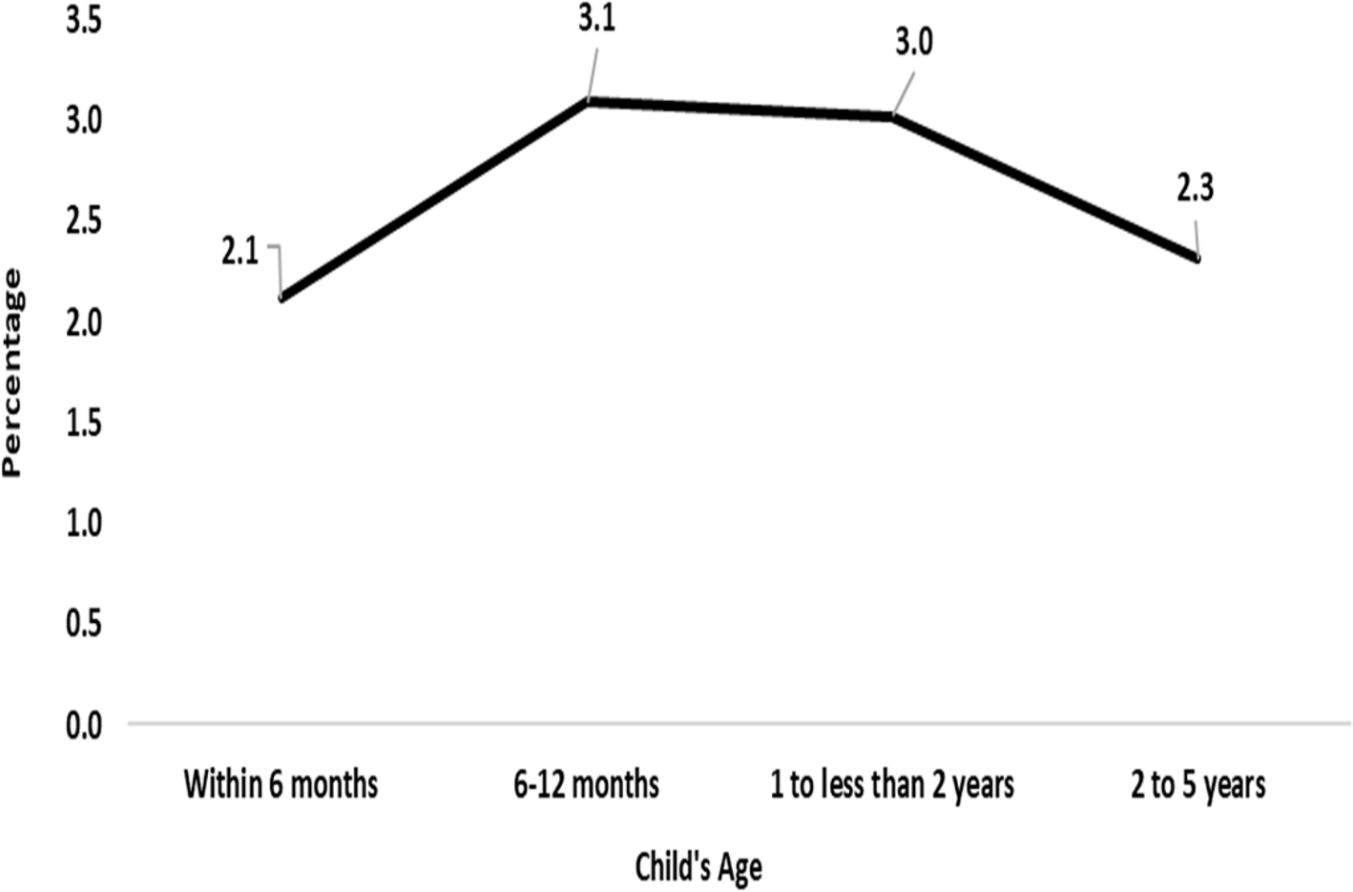
Prevalence of ARI by age groups of under-5 children in North-East region,India,2019–21

Figures 5 and 6, illustrate the prevalence of ARI among children across different age groups in both individual states and the entirety of the northeastern states of India. Notably, Meghalaya shows the highest prevalence of ARI within the 6-month age group. In Meghalaya, the highest prevalence is seen among children aged 6–12 months and those between 1–2 years old, and records the highest rates among children aged 2–5 years. An overarching observation across the entire northeastern region is the heightened prevalence of ARI among children aged 6–12 months.

### For diarrhoea

Table 5 indicates the result of the logistic regression of the factors affecting the diarrhoea among under-5 year children in Northeast Indian states. In Model 1, after adjusting for household, mother and child covariates, the odds of having diarrhoea was 2.6 times more in Meghalaya than in Sikkim. It was statistically highly significant (AOR = 2.66 and *p* < 0.01). If we examined the mother characteristics, for the children of the women who received higher education, the odds of having diarrhoea was 36 percent lesser as comparison to the children of illiterate women. In religion, the children who were born to a Muslim women had 65 percent higher chance of getting diarrhoea as comparison to the children born to a Hindu woman and it was statistically highly significant (*p* < 0.01). The odds of having diarrhoea among children born to a ST woman was 38 percent higher as comparison to Other caste woman (*p* < 0.05) and among children of OBC women the odds of having diarrhoea was 40 percent higher as comparison to Other caste women (*p* < 0.05). The odds of having diarrhoea among children of poorer women was 13 percent lesser as comparison to children of poorest women. Similarly among the household characteristics, the children living in the household with unimproved sanitation facilities had a 16 percent higher chance of getting diarrhoea compared to the household with improved sanitation facilities, which was statistically significant (*p* < 0.05). In child characteristics, as we move from children with 1st birth order to 2nd,3rd and 4 plus birth order, the odds of getting diarrhoea was more likely and statistically highly significant (*p* < 0.01). Similarly, the odds of having diarrhoea was 2.18 times higher among children of 6 to 12 months as comparison to children with age less than 6 months and the odds of having diarrhoea was 96 percent higher among the children aged 1 to less than 2 years as comparison to children with age less than 6 months. Again, the odds of having diarrhoea among children aged 2 to 5 years was 39 percent more as comparison to the children aged less than 6 months and it was statistically highly significant (*p* < 0.01). The odds of having diarrhoea among children who were born by caesarean delivery had 62 percent higher as comparison to the reference category and it was statistically highly significant (*p* < 0.01).In Model 2,when we adjust for programme factors like BCG,DPT and Rota virus in the Model1,the significance level of the covariate remain nearly same and the effect of the programme factors on the childhood diarrhoea was found to be insignificant. The covariates like place of residence, mother’s age, number of children living, child being stunted were insignificant.

**Table 5:** Results of logistic regression of factors affecting the diarrhoea, fever and ARI among children of under 5 years in North east India,2019–21

Variables	Diarrhoea				Fever				ARI			
	**Model 1 (*n* = 26,568)**		**Model2 (*n* = 15,118)**		**Model 1 (*n* = 26,568)**		**Model2 (*n* = 15,118)**		**Model 1(*n* = 26,568)**		**Model2 (*n* = 15,118)**	
	**AOR**	**SE**	**AOR**	**SE**	**AOR**	**SE**	**AOR**	**SE**	**AOR**	**SE**	**AOR**	**SE**
**Community characteristics**												
** State/Region**												
Sikkim **(ref.)**	1		1		1		1		1		1	
Arunachal Pradesh	1.51	0.342	1.58	0.481	0.72**	0.106	0.86	0.17	2.45	1.05	0.86	0.17
Nagaland	1.09	0.269	1.65	0.533	0.75	0.12	0.97	0.207	1.32	0.616	0.97	0.207
Manipur	1.29	0.307	1.52	0.478	0.94	0.141	1.11	0.224	1.8	0.8	1.11	0.224
Mizoram	0.98	0.251	1.25	0.42	0.70**	0.115	0.87	0.188	0.59	0.312	0.87	0.188
Tripura	1.34	0.331	1.55	0.508	1.34	0.21	1.70**	0.362	1.39	0.645	1.70**	0.362
Meghalaya	2.66***	0.613	3.37***	1.029	2.59***	0.38	3.67***	0.724	5.17***	2.23	3.67***	0.724
Assam	1.01	0.234	0.98	0.304	1.3	0.189	1.54**	0.303	1.73	0.743	1.54**	0.303
** Place of Residence**												
Urban **(ref.)**	1		1		1		1		1		1	
Rural	1.09	0.097	1.16	0.135	0.86***	0.049	0.83**	0.062	0.82	0.106	0.83**	0.062
**Mother's characteristics**												
** Age**												
15–24 **(ref.)**	1		1		1		1		1		1	
25–34	0.9	0.063	0.84**	0.072	0.96	0.046	1.02	0.061	1.01	0.115	1.02	0.061
35–49	0.88	0.086	0.84	0.106	0.96	0.064	0.91	0.081	1.01	0.153	0.91	0.081
** Education**												
Illiterate** (ref.)**	1		1		1		1		1		1	
Primary	1.14	0.098	1.17	0.132	1.17**	0.074	1.16	0.095	1.05	0.135	1.16	0.095
Secondary	0.98	0.078	0.96	0.101	1.27***	0.073	1.24***	0.093	1	0.119	1.24***	0.093
Higher	0.64***	0.094	0.63**	0.119	1.05	0.099	0.95	0.115	0.86	0.184	0.95	0.115
** Religion**												
Hindu **(ref.)**	1		1		1		1		1		1	
Muslim	1.65***	0.259	1.77***	0.345	1.27**	0.13	1.26	0.166	1.56	0.398	1.26	0.166
Christian	0.91	0.099	0.77	0.108	1.11	0.085	1.03	0.102	0.76	0.129	1.03	0.102
Others	1.1	0.132	1.01	0.157	1.27***	0.11	1.14	0.131	1.02	0.192	1.14	0.131
** Caste**												
Others **(ref.)**	1		1		1		1		1		1	
SC	1.18	0.173	1.12	0.21	0.78***	0.071	0.83	0.098	2.08***	0.496	0.83	0.098
ST	1.38**	0.176	1.32	0.215	0.76***	0.06	0.77**	0.079	1.88***	0.413	0.77**	0.079
OBC	1.40**	0.185	1.31	0.22	1.14	0.09	1.11	0.115	2.37***	0.517	1.11	0.115
** Wealth Index**												
Poorest **(ref.)**	1		1		1		1		1		1	
Poorer	0.87**	0.056	0.86	0.07	0.97	0.043	0.99	0.056	0.87	0.085	0.99	0.056
Middle	0.86	0.072	0.85	0.091	0.83***	0.049	0.84**	0.063	0.81	0.106	0.84**	0.063
Richer	0.76	0.091	0.71**	0.114	0.84**	0.066	0.83	0.087	0.64**	0.124	0.83	0.087
Richest	0.73	0.153	0.78	0.205	0.89	0.113	1.04	0.167	0.7	0.222	1.04	0.167
**Household characteristics:**												
** Sanitation/Toilet Facilities**												
Improved **(ref.)**	1		1		1		1		1		1	
Not improved	1.16**	0.073	1.11	0.09	1.15***	0.05	1.11	0.062	1.15	0.11	1.11	0.062
** Number of living children**												
One child** (ref.)**	1		1		1		1		1		1	
Two children	0.7	0.076	0.82	0.145	0.63***	0.048	0.79	0.097	0.75	0.134	0.79	0.097
3 or more children	0.48	0.076	0.57**	0.146	0.43***	0.048	0.66	0.117	0.77	0.188	0.66**	0.117
**Child characteristics**												
** Birth Order**												
1 BO **(ref.)**	1		1		1		1		1		1	
2 BO	1.37***	0.151	1.1	0.196	1.40***	0.108	1.07	0.133	1.26	0.224	1.07	0.133
3 BO	1.71***	0.273	1.44	0.365	1.87***	0.209	1.34	0.237	1.2	0.302	1.34	0.237
4 plus BO	1.92***	0.319	1.72**	0.459	2.53***	0.293	1.74***	0.322	1.84**	0.464	1.74***	0.322
** Stunting**												
No **(ref.)**	1		1		1		1		1		1	
Yes	1.04	0.057	0.92	0.067	0.98	0.037	0.96	0.047	1.13	0.096	0.96	0.047
** Child Age**												
within 6 months **(ref.)**	1		1		1		1		1		1	
6–12 months	2.18***	0.274	2.22***	0.299	1.99***	0.167	2.04***	0.187	1.89***	0.366	2.04***	0.187
1to less than 2 years	1.96***	0.238	2.04***	0.275	1.91***	0.154	1.94***	0.176	1.70***	0.317	1.94***	0.176
2 to 5 years	1.39***	0.159	1.37***	0.19	1.36***	0.102	1.40***	0.129	1.34	0.234	1.40***	0.129
** Caesarean delivery**												
No **(ref.)**	1		1		1		1		1		1	
Yes	1.62***	0.122	1.64***	0.157	1.31***	0.069	1.29***	0.087	2.12***	0.231	1.29***	0.087
**Programme factors**												
** BCG vaccine**												
No **(ref.)**	–-		1		–-		1		–-		1	
Yes			1.09	0.121			1.14	0.088			1.14	0.088
**DPT vaccine**												
No **(ref.)**	–-		1		–-		1		–-		1	
Yes			0.97	0.097			0.99	0.069			0.99	0.069
**Rota virus vaccine**												
No** (ref.)**	–-		1		–-		1		–-		1	0.057
yes			1.08	0.104			0.87**	0.057			0.87**	
** Constant**	0.02***	0.007	0.02***	0.009	0.11***	0.021	0.08***	0.022	0.005***	0.003	0.08***	0.022

*** means *p* < 0.01; ** means *p* < 0.05

### For fever

Table 5 shows the Adjusted odds ratio (AOR) from the logistic regression to examine the factors affecting fever among under 5 year children in Northeast Indian states. Results from Model 1 showed that the in comparison to the fever among children in Sikkim, the odds of fever in Arunachal Pradesh was 28 percent less and in Mizoram it is 30 percent less respectively and it was statistically significant (*p* < 0.05). In Meghalaya the odds of fever is 2.59 times higher compared to the fever among children in Sikkim, which was statistically highly significant (*p* < 0.01). In rural areas, the odds of having fever was lower compared to urban areas (AOR = 0.86, *p* < 0.01). The odds of having fever among children of the women who received primary or secondary education was higher in comparison to the illiterate women. Muslim children (AOR = 1.27, *p* < 0.05) and the children of other religion (AOR = 1.27, *p* < 0.01) were more likely to get fever in comparison to Hindu children. Among the caste group, the odds of having fever was lower among the children of SC (AOR = 0.78,*p* < 0.01) and ST women (AOR = 0.76, *p* < 0.01) in comparison to children of Other caste group women. The children belonged to the middle and richer wealth Index, the odds of having fever was lower than the poorest wealth index. The chance of having fever among children living in a household with unimproved sanitation was 15 percent higher compared to the children living in a household with improved sanitation facilities. It was statistically highly significant (*p* < 0.01). Compared to the household with 1 child, the odds of having fever in the two children household was 37 percent lesser and for 3 or more children household it was 57 percent lesser and the result was statistically highly significant (*p* < 0.01). For Birth order of the children, the odds of having fever was 40 percent higher for 2nd birth order children and 87 percent higher for 3rd birth order children compared to 1st birth order children. It was statistically highly significant (*p* < 0.01). The odds of having fever among 4 plus birth order children was 2.53 times higher compared to 1st birth order child (*p* < 0.01). Among the children of different ages, the odds of having fever was respectively 99 percent higher (*p* < 0.01) among 6 to 12 months old children,91 percent higher(*p* < 0.01) for 1 to less than 2 year old children and 36 percent higher (*p* < 0.01) for 2 to 5 years old children in comparison to the children of less than 6 months. The children who were born out of caesarean delivery, the odds of having fever was 31 percent higher in comparison to the reference category and it was statistically highly significant (*p* < 0.01). When we added programme factors in Model 1,in the Model 2, the odds of having fever was higher for Tripura, Meghalaya and Assam than Sikkim. Other covariates of the model showed the similar statistically significant relationship as in Model 1. However, among the programme factors, the children who received Rota virus vaccine, the chance of having fever among children was 13 percent lesser than those who didn’t receive. In Model 1, mother’s age, and child being stunted was insignificantly affecting fever among children and In Model 2, factors like mother’s age, religion, sanitation facilities, number of living children, stunting, BCG and DPT vaccination were insignificantly affecting fever among children.

### For ARI

Table 5 showed the Adjusted odds ratio (AOR) from the logistic regression to examine the factors affecting ARI among under 5-year children in Northeast Indian states. From Model 1,we found that in the state of Meghalaya the children had 5.17 times more chance of getting ARI than the children of Sikkim, which was statistically highly significant (*p* < 0.01). The chances of getting ARI among children belonging to the SC caste group was 2.08 times higher in comparison with children of Other caste group. Similarly, in comparison with children of Other caste group, the chances of getting ARI was 88 percent more among children of ST caste group and 2.37 higher among OBC children respectively and the results were statistically highly significant (*p* < 0.01). For the children who belong to richer wealth category, the odds of getting ARI was 36 percent lower compared to children from the poorest wealth category (*p* < 0.05). For the birth order, the children who belonged to 4 or higher birth order had a higher chance of getting ARI than 1st birth order children (AOR = 1.84, *p* < 0.05). If we examine the child’s age, in comparison to the children aged less than 6 months, the odds of getting ARI was 89 percent higher for children of 6 to 12 months and 70 percent more for children of the 1 to 2 year age respectively. The children who were born out of caesarean delivery, the chances of getting ARI was 2.12 times higher as comparison to those children who was not delivered by caesarean and the result was statistically highly significant (*p* < 0.01). If we add programme factors like BCG, DPT and Rota virus in Model 1, in Model 2, the results showed that the chances of getting ARI was higher in Tripura, Meghalaya and Assam than in Sikkim state. The rural children had lower chance of ARI than urban children. The children with secondary education had higher chance of getting ARI in comparison to illiterate women children. Other factors were similarly affecting the ARI among children as in Model 1. Among the programme factors, the chances of getting ARI was 13 percent lower among those who received Rota virus than those who did not. The BCG and DPT vaccination was insignificantly affecting ARI. In Model 1, place of residence, mother’s age, mother’s education, religion, sanitation facilities, number of living children and child being stunted was insignificantly affecting ARI among children. In Model 2, the insignificant factors were mother’s age, religion, sanitation facilities, child being stunted. Some of the highlights of the important findings emerging from previous and current research has been shown in Table 6 as ready reckoner.

**Table 6:** Highlights of findings emerged from the present study on the illnesses of Diarrhoea, Fever and ARI in Northeast India,2019–21

*Known about the illnesses (Diarrhoea, Fever & ARI) in India & Northeast from previous studies*	*New emerging points from our study about the illnesses (Diarrhoea, Fever & ARI) in the 8 states* *of Northeast India*
• A study using NFHS-4 (2015–16) data revealed that the north-east region as a reference, those from north, central and east regions were more likely to suffer from ARI. Comorbidity, sex, age and nutritional status of children were significantly associated with the prevalence of ARI [51] • It is found that among children in northeast India, the overall incidence of fever remains more or less unchanged but that of cough and diarrhoea have declined considerably during 2004–05 to 2011–12.Girls are less likely to have suffered from diarrhea than the boys; age has a significant effect on the risk of children suffering from fever and cough, toilet and having access to improved sources of drinking water lower the risk of childhood morbidities [27] • In a multicounty study including India, using NFHS4 data it was found that children who were given the measles vaccine were less likely to suffer from Diarrhoea and ARI in India [52] • Using NFHS-4 data, the study results suggests that most districts situated in India’s north and central regions had higher chances of ARI and diarrhoea and cases of diarrhoea may reduce with the improved toilet facilities, female children are less prone to ARI and Diarrhoea; stunted and wasted children are more susceptible to Diarrhoea only; young women with low education level are more likely to have children down with both the diseases, Hindu and ST have less while SC children have more chances of being sick with ARI and diarrhoea [20] • Using NFHS-4 data, the prevalence of diarrhoea and ARI was 13.8% and 3.4% among children in India. Association was observed between exclusive breastfeeding with Diarrhoea and ARI [53] • Using NFHS-4 data, prevalence of diarrhoea in India among under 5 children is higher in rural areas, not staying in Pacca house, living with unimproved sanitation facilities, belonging to under privileged community, children of younger mothers and “poor’ households [16] • Using NFHS-5 data (2019–21) a study on spatial clustering of diarrhoea in India among children under 5 years found that the Prevalence of diarrhoea in Meghalaya (10.5%), is third highest in India, with an increase in children’s age as well as mother's age the prevalence of the diarrhoea decreases. prevalence is more among male children than females, Underweight children have a greater risk of suffering from diarrhoea diseases, the odds of children living in a pucca house are less likely to suffer from diarrhoea. rich economic status reduces the risk of such morbid conditions [2]	***From NFHS-5 (2019–21) data revealed*** *About Diarrhoea* • Prevalence of diarrhoea in Meghalaya (10%), Tripura (6%), Assam, Arunachal Pradesh, Sikkim, Manipur lied (5–5.5%), Mizoram (4.3%) and least in Nagaland (3.4%) and overall northeast it is 6% • Among northeast states and age wise prevalence, Sikkim has the highest prevalence (18.4%) of diarrhoea among children of less than 6 months, Meghalaya has highest prevalence of diarrhoea for children of age-group 6–12 months and 1–2 years while Arunachal Pradesh has highest rate of diarrhoea among 2–5 years children • In comparison to the children of less than 6 months, the odds of diarrhoea among 6 to 12 months children were 2.18 times more and for 1–2 years children are 96% higher and for 2 to 5 years it is 39 percent higher • The odds of diarrhoea was higher among children born in cesarean delivery than not cesarean delivery • The vaccinations like BCG, DPT and Rota virus was found to have insignificant effect on diarrhoea in northeast states ***About Fever*** • A higher prevalence of fever was observed in Meghalaya (23%) and lowest in Arunachal Pradesh (9%) • Among all age groups of children (less than 6 months,6 to 12 months,1–2 year and 2to 5 years) in northeast Indian states, the prevalence is highest in Meghalaya • Compared to 1st birth order children, the 4 plus birth order children has more than 2 times higher odds of fever • The programme factors like BCG and DPT was insignificant, however the Rota virus vaccination lowers the odds of fever among children • The odds of fever were higher for the children born out of cesarean delivery than not born out of it ***About ARI*** • Higher prevalence of ARI was observed in Meghalaya (4.8%) followed by Assam (2.5%), Arunachal Pradesh (2.1%) while Mizoram and Sikkim it is less than 1 percent • Among all age groups of children (less than 6 months,6 to 12 months,1–2 year and 2 to 5 years) in northeast Indian states, the prevalence of ARI is highest in Meghalaya • The odds of ARI were higher for the children born out of cesarean delivery than not born out of it • In comparison to the children of less than 6 months, the odds of ARI among 6 to 12 months children and for 1–2 years children are higher • The vaccinations like BCG, DPT was insignificantly affecting the ARI, however, there is lower odds of ARI among the children who were given Rota virus than those who were not given

## Discussion and conclusion

This study delved into the socio-demographic and environmental aspects associated with diarrhoea, fever and ARI among children below five years old in India and the factors that might contribute to their occurrence. Despite a decline in the prevalence of childhood diarrhoea in recent times, the substantial burden of this preventable illness persists. We used the NFHS-5 dataset to conduct analyses using bivariate and multivariate methods. It was revealed that 5.95 percent,17.36 percent and 2.51 percent of the children had suffered diarrhoea, fever and ARI respectively, two weeks before the survey. In comparison to Sikkim, state of Meghalaya was found to have more diarrhoea, ARI and fever and it was statistically highly significant, however, Tripura and Assam had significant higher odds of having fever and ARI in comparison to Sikkim. After controlling for programme factors (mainly Rota virus) in model 2, the lower odds of having fever in Arunachal Pradesh and Mizoram were found to disappear from the previous model, which shows the impact of Rota virus on the fever among children under 5 years. Results also reveals that these Illnesses more influence the male child.There are some studies in northeast India and other parts of India and world which also found male to be more susceptible to the illnesses than female [27, 54].

The findings of this study indicated that children residing in rural regions were at a higher likelihood of encountering diarrhoea, fever, and ARI compared to those in urban areas which is same in the previous study conducted in India [8]. Clear disparities based on region and location were noticeable, showcasing increased vulnerability to the presence of diarrhoea, fever, and ARI in the northern states region and rural areas [42, 45].

In this study, caste and religion displayed notable associations with childhood diarrheal illnesses. Specifically, the research identified that children from scheduled tribes and other caste groups exhibited a reduced risk of diarrhea in comparison to those from scheduled castes. This discovery aligns with similar findings from prior studies conducted in India [55].

This study demonstrated a significant correlation between the household's wealth status and recent diarrhea among children. The research findings highlighted a decreased probability of diarrhea in children from the wealthiest wealth quintile compared to those in the poorest. A similar study in Peru has the same contribution [9]. Thus, the government's initiatives to elevate the incomes of impoverished individuals through the execution of the objectives outlined in the National Development Plan (2010- 15) serve as a crucial means toward achieving Millennium Development Goals MDG 4. The odds of having diarrhoea among children who were born by caesarean delivery had higher as comparison to the home or normal delivery. Similar findings was also found in the previous studies where the reported c-section was associated to the risk of gastroenteritis among children aged > 1 year and also less than 1 year [56–58].

Our findings shows that those who have unimproved sanitation facilities is highly associated with diarrhoea and fever. This outcome aligns with findings from a study in India [3, 46].

According to the findings of this study, there exists an association between the occurrence of diarrhoea, fever, and ARI and factors such as the age of the child and caregiver, the wealth status of the household, the quality of sanitation facilities, methods of stool disposal, and the educational level of the caregiver. It suggests directing efforts toward implementing programs that aim to decrease diarrhoea and ARI among children under five by addressing socioeconomic barriers that hinder caregivers' access to wealth and education. The window of opportunity to prevent the co-occurrence of diarrhoea, fever, and ARI in children under 5 years old is crucial during the age range of 2 to 5 years. The window of opportunity to prevent these childhood illnesses relates to the proper nutrition and follow of timely immunisation schedule, adequate water and sanitation facilities, hygiene, well ventilated houses and no indoor pollution,proper stool disposal of children [8, 59, 60]. Consequently, tailored health education and promotional initiatives targeting caregivers or mothers from disadvantaged backgrounds, having lower educational levels, and are younger should be devised to prevent these illnesses in the area. Moreover, additional interventions focusing on enhancing water sources, sanitation, and family planning strategies should also be developed.

## Data Availability

The link for the publicly available free dataset is mentioned below https://dhsprogram.com/
